# Colorectal cancer DNA methylation patterns from patients in Manaus, Brazil

**DOI:** 10.1186/s40659-015-0042-7

**Published:** 2015-09-12

**Authors:** Fabiana Greyce Oliveira Almeida, Priscila Ferreira de Aquino, Afonso Duarte Leão de Souza, Antonia Queiroz Lima de Souza, Sonia do Carmo Vinhote, Thaís Messias Mac-Cormick, Marcelo Soares da Mota Silva, Sidney Raimundo Silva Chalub, Juliana de Saldanha da Gama Fischer, Paulo Costa Carvalho, Maria da Gloria da Costa Carvalho

**Affiliations:** Laboratory of Chromatography and Mass Spectrometry, Institute of Exact Sciences, Federal University of Amazonas, Manaus, Brazil; Leonidas and Maria Deane Institute, FIOCRUZ, Manaus, Brazil; High School of Health, Amazon State University, Manaus, Brazil; Laboratory of Molecular Pathology, Department of Pathology, Federal University of Rio de Janeiro, Rio de Janeiro, Brazil; Department of Abdominal Surgery, Oncology Control Center Foundation of Amazon State, Manaus, Brazil; Laboratory of Proteomics and Protein Engineering, Carlos Chagas Institute, Curitiba, Brazil

**Keywords:** Methylation, Colorectal cancer, Amazon, Epigenetic

## Abstract

**Background:**

DNA methylation is commonly linked with the silencing of the gene expression for many tumor suppressor genes. As such, determining DNA methylation patterns should aid, in times to come, in the diagnosis and personal treatment for various types of cancers. Here, we analyzed the methylation pattern from five colorectal cancer patients from the Amazon state in Brazil for four tumor suppressor genes, viz.: *DAPK*, *CDH1*, *CDKN2A*, and *TIMP2* by employing a polymerase chain reaction (PCR) specific to methylation. Efforts in the study of colorectal cancer are fundamental as it is the third most of highest incidence in the world.

**Results:**

Tumor biopsies were methylated in 1/5 (20 %), 2/5 (40 %), 4/5 (80 %), and 4/5 (80 %) for *CDH1*, *CDKN2A*, *DAPK*, and *TIMP2* genes, respectively. The margin biopsies were methylated in 3/7 (43 %), 2/7 (28 %), 7/7 (100 %), and 6/7 (86 %) for *CDH1*, *CDKN2A*, *DAPK*, and *TIMP2*, respectively.

**Conclusions:**

Our findings showed *DAPK* and *TIMP2* to be methylated in most samples from both tumor tissues and adjacent non-neoplastic margins; thus presenting distinct methylation patterns. This emphasizes the importance of better understanding of the relation of these patterns with cancer in the context of different populations.

## Background

Colorectal cancer (CRC) comprehends tumors that affect the colon and rectum; it is the third type of cancer with the highest incidence in the world [1]. According to the 2014 estimates for Brazil, about 576,000 new cases of cancer were expected [2] of which 15,070 of those being for CRC in men and 17,530 for women. In particular, for the Amazon state (Brazil), a rate of 4.65 cases per 100,000 inhabitants [2] was estimated. The standard treatment for localized colorectal cancer involves surgical resection by open surgery of the primary tumor and regional lymph nodes. It has been shown that excessive consumption of red meat, alcoholism, body and abdominal fat, smoking, family history of CRC, genetic susceptibility to the development of chronic intestine disease, and age are all positively correlated to CRC [3, 4]. Nevertheless, about 90 % of CRC occurs sporadically and without family history or genetic predisposition; i.e., less than 10 % of the cases are believed to be linked to one’s genetics [5].

The study of epigenetics alterations has gained increasing attention; it comprises investigating how external or environmental factors affect the control of gene expression by turning genes “on” or “off” [6]. An example of a key epigenetic event is the methylation that occurs in small regions of DNA called CpG island which are located in the promoter region of genes [7]. Methylation defines the process of adding a methyl (CH_3_–) group in the promoter cytosine bases at the 5′ position to form 5-methylcytosine (5-MC). DNA methylation is usually linked with the silencing of gene expression for many tumor suppressor genes, such as, *CDH1* [8], *CDKN2A* [9], *TIMP2* [10], and *DAPK* [11]. The *CDH1* gene expresses E-cadherin which is a transmembrane protein contained between epithelial cells and represents one of the main proteins involved in cell adhesion [12]. The loss of adhesion mediated by E-cadherin appears to have a fundamental importance in neoplastic processes. A down regulation of this gene is correlated with a decrease in the efficiency of cellular adhesion and therefore facilitating cellular motility; this in turn facilitates cancer cells for invading surrounding tissues [8]. Another gene strongly correlated to cancer is the *CDKN2A*; one of its products is the p16, a regulatory protein that inhibits the progression of cells through the G1 phase of the cell cycle [11]. Inactivation of p16 can cause abnormal cells and uncontrolled cell growth [13]. The *TIMP2*, for tissue inhibitor of metalloproteinases 2, is known to antagonize the activity of matrix metalloproteinases and suppress tumor growth, angiogenesis, invasion, and metastasis [14]; the mechanism of inhibition of this gene’s expression remains unknown. Finally, *DAPK* (for death-associated protein kinase) participates in several functions in the cell, among them, playing a key role in the reorganization of the cytoskeleton in cytokine stimulation and induction of apoptosis [11]. Understanding *DAPK*’s role in transcriptional regulation may lead to the discovery of novel therapeutics to combat cancer and inflammation associated diseases.

According to literature, abnormal methylation patterns of these genes is an indicative of cancer [15–18]. Studies have reported such alterations, for example, in the mucosa of colorectal tumor and suggested these genes as cancer markers [19, 20]. In another work, the hypermethylation of *DAPK* and *CDKN2A* in the normal colonic mucosa of patients with CRC was evaluated by the methylight assay and the results showed their levels of methylation to be relatively low [mean percentage of a methylated reference (PMR) <1] [20]. In the same work, the authors correlated patients with high levels of methylation of *INK4A* and *DAPK* with advanced age (p < 0.1), supporting the hypothesis that age is one of the risk factors for this pathology. Finally, a panel of methylation markers for CRC diagnosis comprising *CDKN2A*, *MGMT*, *MLH1*, and *SFRP* was proposed [21].

Even though epigenetic markers are being increasingly applied in the screening for colorectal neoplasia’s samples, further investigations, especially in different populations, is fundamental for a proper understanding of the applicability of these markers to the clinical practice. In the present study, we evaluated the methylation of the *CDH1*, *DAPK*, *CDKN2A*, and *TIMP2* genes in five colorectal cancer patients from Manaus, the capital of the Amazon state in Brazil. Although the Amazon region presents a predominance of Indians, with approximately 342,800 indigenous people [22], this study included only patients recognized as having brown or white skin. This is the first study of this type performed in the population of the Amazon region.

## Results

Table 1 shows the clinical characteristics of the five patients included in this study; we note that 20 % (1/5) of the tumors were located in colon and 80 % (4/5) in the rectum. The median age of the patients was 62 years. All the patients had resection margins free of neoplasia according to a histopathological exam. These results were compared to the methylation status obtained here. All of the tumor-node-metastasis (TMN) stages analyzed here had one or more genes methylated. Table 2 shows the gene methylation status for the *CDH1*, *CDKN2A*, *DAPK*, and *TIMP2* genes.

**Table 1:** Patients’ clinical characteristics

Patients no/sample	Cancer type	Gender	Age (years)	Skin color	Stage (TMN)
4	Rectal adenocarcinoma	M	65	Brown	T3N0M0
7	Colon adenocarcinoma	M	40	Brown	T3N0M0
8	Rectal adenocarcinoma	F	74	Brown	T4N2M0
1	Rectal adenocarcinoma	M	61	Brown	T3N0M0
9	Rectal adenocarcinoma	M	62	White	T3N1M0

*M* male, *F* female, *TMN* tumor-node-metastasis

**Table 2:** Methylation status of *CDH1*, *CDKN2A*, *DAPK* and *TIMP2* genes

Patient no/sample	Methylation			
	*CDH1*	*CDKN2A*	*DAPK*	*TIMP2*
4 T	M	M	M	M
4 PM	M	M	M	M
4 DM	U	M	M	M
7 T	U	U	U	M
7 PM	M	U	M	M
7 DM	M	U	M	M
8 T	U	U	M	M
8 DM	U	U	M	M
1 T	NA	M	M	NA
1 DM	U	U	M	NA
9 T	U	U	M	M
9 DM	NA	U	M	M
Methylation tumor (%)	1/5 (20 %)	2/5 (40 %)	4/5 (80 %)	4/5 (80 %)
Methylation adjacent margin (%)	3/7 (43 %)	2/7 (28 %)	7/7 (100 %)	6/7 (86 %)

*NA* not amplified, *U* unmethylated, *M* methylated, *T* tumor, *PM* proximal margin (2 cm), *DM* distal margin (5 cm)

Tumor biopsies were methylated in 1/5 (20 %), 2/5 (40 %), 4/5 (80 %), and 4/5 (80 %) for the *CDH1*, *CDKN2A*, *DAPK*, and *TIMP2* genes, respectively. The margin biopsies were methylated in 3/7 (43 %), 2/7 (28 %), 7/7 (100 %), and 6/7 (86 %) for the *CDH1*, *CDKN2A*, *DAPK*, and *TIMP2* genes, respectively. In all, 64 % (18/28) of the resection margins and 55 % (11/20) of tumor tissue were methylated for the four genes.

Patient 4 had the tumor biopsy (4T) and margins (4PM and 4DM) methylated for all, *CDH1*, *CDKN2A*, *DAPK*, and *TIMP2*, except for 4DM not being methylated for *CDH1*. The tumor tissue of patient 8 (8T) and the respective adjacent margin (8DM) were not methylated for *CDH1* and *CDKN2A*; yet, *DAPK* and *TIMP2* were both methylated for 8T and 8DM. These results were similar to those from patient 9DM; other sections from this patient, in our hands, were unable to have their genes amplified by PCR. Patient 1 showed 1T to be methylated for *CDKN2A* and *DAPK* and not for *CDH1* and *TIMP2*. *DAPK* showed to be methylated for 1DM. 7T was not methylated for 7DM but was methylated for both *DAPK* and *CDH1* and thus showing a different methylation pattern. Amplification of *CDH1* gene (1T, 6T, and 9DM samples) and *TIMP2* (sample 1DM) were unsuccessful and methylation could not be analyzed.

## Discussions

The DNA methylation leads to an altered gene expression resulting in changes in the control of cell proliferation and therefore being recognized as a major epigenetic modification in human genes [19]. There is increasing evidence that an aberrant methylation pattern is actively involved in early carcinogenesis [7]. In colorectal cancer, both hyper- and hypomethylation have been observed across different stages of progression. Here, we evaluated the prevalence of hypermethylation in the *CDH1*, *CDKN2A*, *DAPK*, and *TIMP2* suppressor tumors genes of colorectal tumors tissue and their non-neoplastic adjacent margin using methylation specific PCR (MSP), which is a highly sensitive and specific technique [23].

Subjects having either brown or white skin were eligible for participating in this study. We note that the brown classification decurrently from the so called *caboclo*s, descendants of Indians and Europeans—mostly English, French, and Spanish, that are commonly found in the northern part of Brazil [24]. It is the first time a study addressing the methylation pattern in colorectal cancer patients from the Amazon is performed. Our results show that each individual presented a distinct panel of methylated genes. In general, the methylated tumor tissues had their respective margin methylated. Likewise, genes, not methylated in tumor tissues were not methylated in their adjacent margin. Yet, patient 7 showed an opposing result by not presenting methylation on the tumor for genes *DAPK* and *CDH1*; these genes were methylated only in the respective adjacent margins (7PM and 7DM). We hypothesize that this may have happened considering that methylation is a reversible process [25] and, in this way, the gene could have been previously methylated in the tumor but metabolic factors reversed it. Another probable explanation is that tumor cells have a great heterogeneity [26] and, therefore, the examined area was not methylated.

The literature provides several studies that compare the methylation status of histologically normal mucosa with the neoplastic tissues. While some show no correlation between the methylation with the pathology, many studies, from around the world, were able correlate methylation of the tumor region and the corresponding adjacent margin [27–30]. Li et al. reported that 2.4 and 57.4 % of the samples were methylated in their tumor and adjacent tissue and that no methylation was detected in all the control tissue. A meta-analysis study revealed the frequency of the *CDH1* promoter methylation in CRC tissues to be higher than those in control tissues (OR = 2.61, 95 % CI = 1:24–5:50, p = 0.012) [31]. When the ethnicity factor was considered, the *CDH1* promoter methylation showed to be closely linked to the pathogenesis of CRC among Asians and Africans, but not among Caucasians. Our results show that the *CDH1* promoter methylation in tumor tissue was lower than in the adjacent margins. *CDH1* gene promoter methylation may lead to aberrant expression of E-cadherin [32]. The loss of adhesion mediated by E-cadherin appears to have a fundamental importance during the neoplastic processes, which allows cells having a controlled stop normal growth signaling, resulting in the loss of differentiation and increase in cell proliferation associated with the invasive behavior [8].

Xing et al. showed that hypermethylation of the *CDKN2A* gene correlated with a poor prognosis for CRC in European and Asian patients [28], but not for patients from other locations. In this regard, Xing et al., highlights the importance of more studies in different geographical locations to determine and clarify whether such suppressor genes serve as a prognostic factor for patients with CRC. In our study, the percentage of methylation for *CDKN2A* was 2/5 (40 %) for tumors tissue and 2/7 (28 %) for adjacent margins. Kuan et al. showed that advanced stage CRC patients presenting *CDKN2A*/p16 methylation were associated with higher risk of CRC recurrence as compared to those with tumor tissues that were not methylated [30].

In the literature, the methylation status of *TIMP2* and *DAPK* is far from a global picture. Here, 80 % (4/5) of tumor samples and 86 % (6/7) of the adjacent margins, respectively, were methylated for *TIMP2*. However, in a histologically exam all margins were diagnosed as being free of tumor. For *DAPK*, 80 % of tumor samples and 100 % of the margins samples were methylated. *S*ince *DAPK* is methylated, its anti-metastatic function is presumed to be lost [33]. Therefore, this work is aligned with the thesis that *DAPK* methylation poses as an important marker for the invasion, metastasis, and apoptotic processes in colorectal neoplasias and thus, ultimately, a potential marker for diagnosis and prognosis.

Even though epigenetic markers are being increasingly applied in the screening for colorectal neoplasia samples, there is still need for further investigations, especially in different populations, such as in Manaus. Although the number of patients in this report is small to draw solid conclusions on the importance of these markers in the context of Manaus population, the *TIMP2* and *DAPK* were methylated on almost all tumors. Furthermore, our results showed a correlation with previous works, thus, supporting our claims.

## Conclusion

Taken together, our results disclose methylation patterns for the *CDH1*, *CDKN2A*, *DAPK*, and *TIMP2* genes on tumors and their respective margins. Although this report was limited to five patients, our results can serve as building blocks for future studies comprising meta-analysis of these genes and thus ultimately contributing to generating models with more statistical power. Such large-scale studies should consider establishing panels tailored towards specific population. Our observations pinpointed the heterogeneity of specific molecular abnormalities in each patient; most noticeably, the *TIMP2* and *DAPK* genes were methylated in almost all samples of tumor and their adjacent margins. Yet, adjacent margins were diagnosed as disease-free according to the histopathological assessment. These facts make evident that a better understanding of these methylation patterns are fundamental, in time to come, to aid in developing more effective treatments and premature diagnosis of colorectal cancer.

## Methods

### Patients

**Fig. 1:**
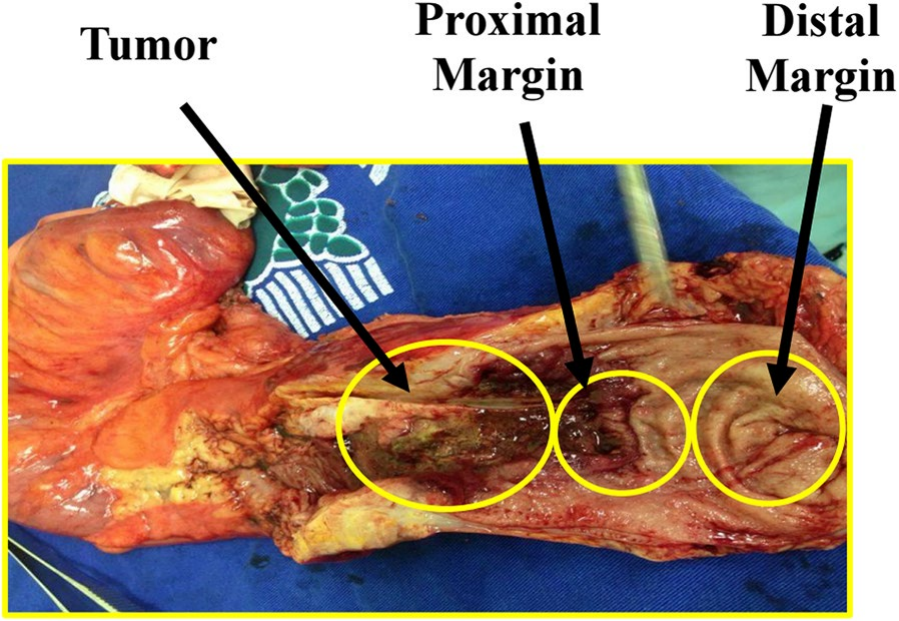
Colorectal cancer tissue obtained after surgery. The tissue was separated into tumor, proximal margin (2 cm), distal margin (5 cm)

This study was approved by the Ethics Committee of the Federal University of Amazonas (UFAM: MEMO, No. 27598614.1.0000.5020, CAAE). The samples were collected at the Oncology Control Center Foundation of Amazonas State (FCECON). After signing informed consent, five patients diagnosed with adenocarcinoma were evaluated. We obtained biopsies of the tumor (T), distal margin (DM), and proximal margin (PM) from two patients (Fig. 1) and only T and DM from the remaining three. Taken together, there were 12 biopsies. These samples were stored at −80 °C. The study includes patients of both genders, all aging between 18 and 80 years and presenting CRC diagnosed by colonoscopy and biopsy.

### DNA extraction

The tissues were pulverized with liquid nitrogen and the DNAs were extracted from each sample using the *kit InnuPrep Forensic* (Biometra, Germany) according manufacturer’s recommendations. The DNAs were quantified using NanoDrop 2000 (Thermo, USA).

### Sodium bisulfite modification

Two micrograms of DNA were used to a final volume of 20 µL in sodium bisulfite modification according the recommendations of the *EZ DNA Methylation*-*Lightning kit manufacturer* (Synapse, Germany). Briefly, 130 µL of lightning conversion reagent were added to 20 µL containing DNA, vortexed and incubated at thermocycler (Mastercycler Gradient, Eppendorff) under the conditions: 98 °C for 8 min, 54 °C for 60 min and 4 °C for 10 min to finish. After transferring the sample to the column, we added 600 µL of M-binding buffer, mixed and centrifuged for 30 s at 10,000×*g*. The supernatant was discarded, 100 µL of M-washing buffer were added, centrifuged again at 10,000×*g* for 30 s, and added to 200 µL of buffer l-desulphonation. The solution was incubated at room temperature for 20 min and then centrifuged at 10,000×*g* for 30 s. Another washing step was performed with 200 µL of M-washing buffer and then centrifuged at 10,000×*g* for 30 s. The modified DNA was eluted from the column by adding 13 µL of M-elution buffer.

### Methylation-specific PCR (MSP)

DNA from the biopsies was subjected to bisulfite treatment and later was amplified by MSP, using primers specific to distinguish methylated from unmethylated DNA in bisulfite-modified DNA, taking advantage of the sequence differences resulting from bisulfite modification. The treatment with bisulfite results in conversion of unmethylated cytosines to uracil and leaves methylated cytosine intact. To detect these changes, specific primers were used (Table 3), as described previously [34–37].

**Table 3:** Oligonucleotides used in the MSP

Primer pair	Methylated set (5′–3′) upstream/downstream	Unmethylated set (5′–3′) upstream/downstream	References
*TIMP2*	5′-AATAAAATTGCGGTTCGGTTTAAGTTC-3′ 5′-CTCTCCTCTTTATCTCGAAAACGCG-3′	5′-GTAATAAAATTGTGGTTTGGTTTAAGTTT-3′ 5′-TTCTCTCCTCTTTATCTCAAAAACACA-3′	[38]
*CDKN2A*	5′-TTATTAGAGGGTGGGGCGGATCGC-3′ 5′- GACCCCGAACCGCGACCGTAA -3′	5′-TTATTAGAGGGTGGGGTGGATTGT-3′ 5′CAACCCCAAACCACAACCATAA-3′	[37]
*DAPK*	5′-GGATAGTCGGATCGAGTTAACGTC-3′ 5′-CCCTCCCAAACGCCGA-3′	5′-GGAGGATAGTTGGATTGAGTTAATGTT-3′ 5′-CAAATCCCTCCCAAACACCAA-3′	[35]
*CDH1*	5′- GTGAATTTTTAGTTAATTAGCGGTAC-3′ 5′-CATAACTAACCGAAAACGCCG-3′	5′- GTAGGTGAATTTTTAGTTAATTAGTGGTA3′ 5′-ACCCATAACTAACCAAAAACACCA-3′	[36]

Oligonucleotides were used for regions containing frequent cytosines (to distinguish between modified and unmodified DNA) and contained CpG dinucleotides at the 3′ end (to provide maximal discrimination between methylated and unmethylated DNA). For each row, the gene names are listed in the primer pair column viz.: *TIMP2* tissue inhibitor of metalloproteinases, *CDKN2A* cyclin dependent kinase 2a/p16, *DAPK* death-associated protein kinase, *CDH1 Cadherin 1*

For PCR analysis, 1 µL of bisulfite modified DNA in a final volume of 12.5 µL of reaction mixture was used containing 1× PCR buffer, MgCl_2_ 1.25 mM, 0.2 mM dNTPs, 0.4 M of each primer, 0.3U Taq polymerase (5U/µL). The reactions methylated and unmethylated were performed in a mastercycler thermocycler (Eppendorff) and the parameters were: 96 °C for 1 min followed by 35 cycles of 94 °C for 1 min, 55 °C for 1 min, 72 °C for 1 min and final extension step of 72 °C for 7 min. The PCR product (10 µL) was directly loaded onto 10 % polyacrylamide gel stained with silver. Negative controls used in PCR were reaction mixture without DNA.
